# Efficient production of d-lactate from methane in a lactate-tolerant strain of *Methylomonas* sp. DH-1 generated by adaptive laboratory evolution

**DOI:** 10.1186/s13068-019-1574-9

**Published:** 2019-09-30

**Authors:** Jong Kwan Lee, Sujin Kim, Wonsik Kim, Sungil Kim, Seungwoo Cha, Hankyeol Moon, Dong Hoon Hur, Seon-Young Kim, Jeong-Geol Na, Jin Won Lee, Eun Yeol Lee, Ji-Sook Hahn

**Affiliations:** 1 School of Chemical and Biological Engineering, Institute of Chemical Processes, Seoul National University, 1 Gwanak-ro, Gwanak-gu, Seoul, 08826 Republic of Korea; 2 Department of Chemical and Biomolecular Engineering, Sogang University, 35 Baekbeom-ro, Mapo-gu, Seoul, 04107 Republic of Korea; 3 Department of Chemical Engineering, Kyung Hee University, 1732 Deogyeong-daero, Giheung-gu, Yongin, 17104 Republic of Korea; 4 Personalized Genomic Medicine Research Center, KRIBB, 125 Gwahag-ro, Yuseong-gu, Daejeon, 34141 Republic of Korea

**Keywords:** Adaptive laboratory evolution, d-Lactate, LysR-type transcriptional regulator, Methane, *Methylomonas* sp. DH-1

## Abstract

**Background:**

Methane, a main component of natural gas and biogas, has gained much attention as an abundant and low-cost carbon source. Methanotrophs, which can use methane as a sole carbon and energy source, are promising hosts to produce value-added chemicals from methane, but their metabolic engineering is still challenging. In previous attempts to produce lactic acid (LA) from methane, LA production levels were limited in part due to LA toxicity. We solved this problem by generating an LA-tolerant strain, which also contributes to understanding novel LA tolerance mechanisms.

**Results:**

In this study, we engineered a methanotroph strain *Methylomonas* sp. DH-1 to produce d-lactic acid (d-LA) from methane. LA toxicity is one of the limiting factors for high-level production of LA. Therefore, we first performed adaptive laboratory evolution of *Methylomonas* sp. DH-1, generating an LA-tolerant strain JHM80. Genome sequencing of JHM80 revealed the causal gene *watR*, encoding a LysR-type transcription factor, whose overexpression due to a 2-bp (TT) deletion in the promoter region is partly responsible for the LA tolerance of JHM80. Overexpression of the *watR* gene in wild-type strain also led to an increase in LA tolerance. When d form-specific lactate dehydrogenase gene from *Leuconostoc mesenteroides* subsp. *mesenteroides* ATCC 8293 was introduced into the genome while deleting the *glgA* gene encoding glycogen synthase, JHM80 produced about 7.5-fold higher level of d-LA from methane than wild type, suggesting that LA tolerance is a critical limiting factor for LA production in this host. d-LA production was further enhanced by optimization of the medium, resulting in a titer of 1.19 g/L and a yield of 0.245 g/g CH_4_.

**Conclusions:**

JHM80, an LA-tolerant strain of *Methylomonas* sp. DH-1, generated by adaptive laboratory evolution was effective in LA production from methane. Characterization of the mutated genes in JHM80 revealed that overexpression of the *watR* gene, encoding a LysR-type transcription factor, is responsible for LA tolerance. By introducing a heterologous lactate dehydrogenase gene into the genome of JHM80 strain while deleting the *glgA* gene, high d-LA production titer and yield were achieved from methane.

## Background

Polylactic acid (PLA) is one of the major bio-based biodegradable plastics in current bioplastic market. So far, optically pure d- or l-LA, the monomer of PLA, has been produced by sugar-based microbial fermentation using genetically engineered bacteria or yeasts [1–5]. Recently, methane, a main component of natural gas and biogas, has gained much attention as a next generation feedstock [6–9]. Methane is not only an abundant and low-cost carbon source but also is a greenhouse gas with a very high global warming potential. Therefore, biological conversion of methane to value-added products such as LA might be a promising strategy in terms of both economic and environmental issues [6, 10].

**Fig. 1:**
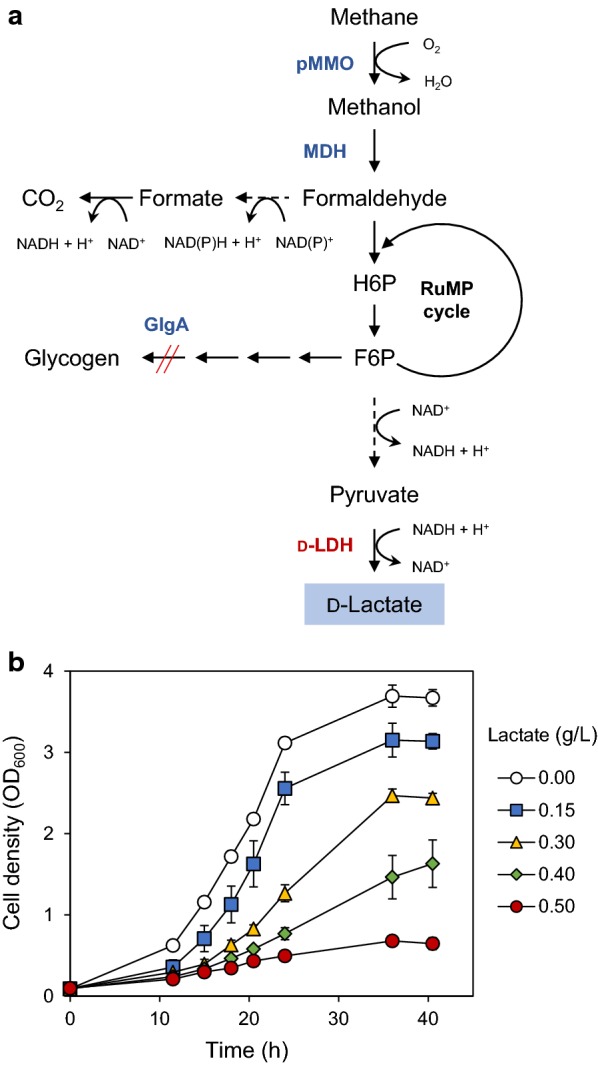
**a** Metabolic pathway for the production of d-LA from methane in *Methylomonas* sp. DH-1. d-LA is produced from pyruvate by heterologous expression of a gene encoding d form-specific lactate dehydrogenase (d-LDH). The *glgA* gene encoding glycogen synthase was deleted to prevent glycogen formation. Dashed arrows indicate multiple pathways. pMMO, particulate methane monooxygenase; MDH, methanol dehydrogenase; H6P, hexulose-6-P; F6P. fructose-6-P; **b** LA tolerance of *Methylomonas* sp. DH-1. Cells were grown in NMS medium supplied with 20% (v/v) methane and the indicated concentrations of LA. The medium pH was adjusted to 6.8. Error bars indicate standard deviations of three independent experiments

Methanotrophic bacteria can grow using methane as a sole carbon and energy source. In aerobic methanotrophs, methane is oxidized to methanol by methane monooxygenase (MMO) and methanol is further oxidized to formaldehyde by methanol dehydrogenase (MDH) (Fig. 1a). Next, formaldehyde is assimilated to biomass thorough 3 different pathways; the Ribulose monophosphate (RuMP) pathway in Group I methanotrophs (Gammaproteobacteria), the Serine cycle in Group II methanotrophs (Alphaproteobacteria), and the Calvin–Benson–Bassham (CBB) cycle in Group III methanotrophs (Verrucomicrobia) [11]. In the RuMP pathway, formaldehyde is converted to fructose-6-phosphate, which then can be converted to pyruvate through Embden–Meyerhof–Parnas (EMP) or Entner–Doudoroff (EDD) pathways (Fig. 1a) [12], making the Group I methanotrophs suitable hosts to produce pyruvate-derived chemicals such as LA. Several genetic manipulation tools have been developed for a few model methanotrophs [13–16], but efficient genetic engineering of many other methanotrophs is still challenging. Moreover, due to the limited understanding of molecular details in the metabolic pathways, metabolic engineering of methanotrophs is currently in a very early stage of development. So far, only a few chemicals such as astaxanthin, butyrate, 2,3-butanediol, succinic acid, and LA were produced using genetically engineered methanotrophs, but with very low titers of less than 1 g/L [17–20].

Previously, LA was produced in *Methylomicrobium buryatense* 5GB1S, a Group I methanotroph, by episomal expression of the lactate dehydrogenase (LDH) gene from *Lactobacillus helveticus*, producing 0.8 g/L l-LA with a yield of 0.05 g/g CH_4_ in continuous gas fermentation [20]. In another study using *M. buryatense* 5GB1 as a host, expression level of the LDH gene was controlled using various promoters and ribosome binding sites, producing 0.5 g/L l-LA in small-scale batch fermentation with periodic methane feeding [21]. Another Group I methanotroph *Methylomicrobium alcaliphilum* 20z^R^ was engineered to increase its endogenous LA production by deleting a gene for pyruvate dehydrogenase, which is involved in a competing pathway of forming acetyl-CoA from pyruvate [22]. However, the LA titer was less than 0.75 g/L under continuous gas fermentation conditions.

In this study, to explore more methanotroph strains available for methane bioconversion, we produced d-LA using Group I methanotroph *Methylomonas* sp. DH-1. *Methylomonas* sp. DH-1, recently isolated from the activated sludge of a brewery plant, has several advantages as a platform strain for methane bioconversion, including fast growth, efficient conversion of methane to methanol, and the availability of annotated genome sequences [23, 24]. Moreover, since *Methylomonas* sp. DH-1 does not have its own LDH gene, this strain is suitable to produce optically pure LA by introducing either d- or l-specific LDH gene. Because LA toxicity can be a limiting factor for efficient production of LA, we developed LA-tolerant mutants of *Methylomonas* sp. DH-1 by adaptive laboratory evolution and generated an efficient d-LA-producing strain by introducing D-specific LDH gene into the evolved strain.

## Results

### Development of LA-tolerant strains by adaptive laboratory evolution

**Fig. 2:**
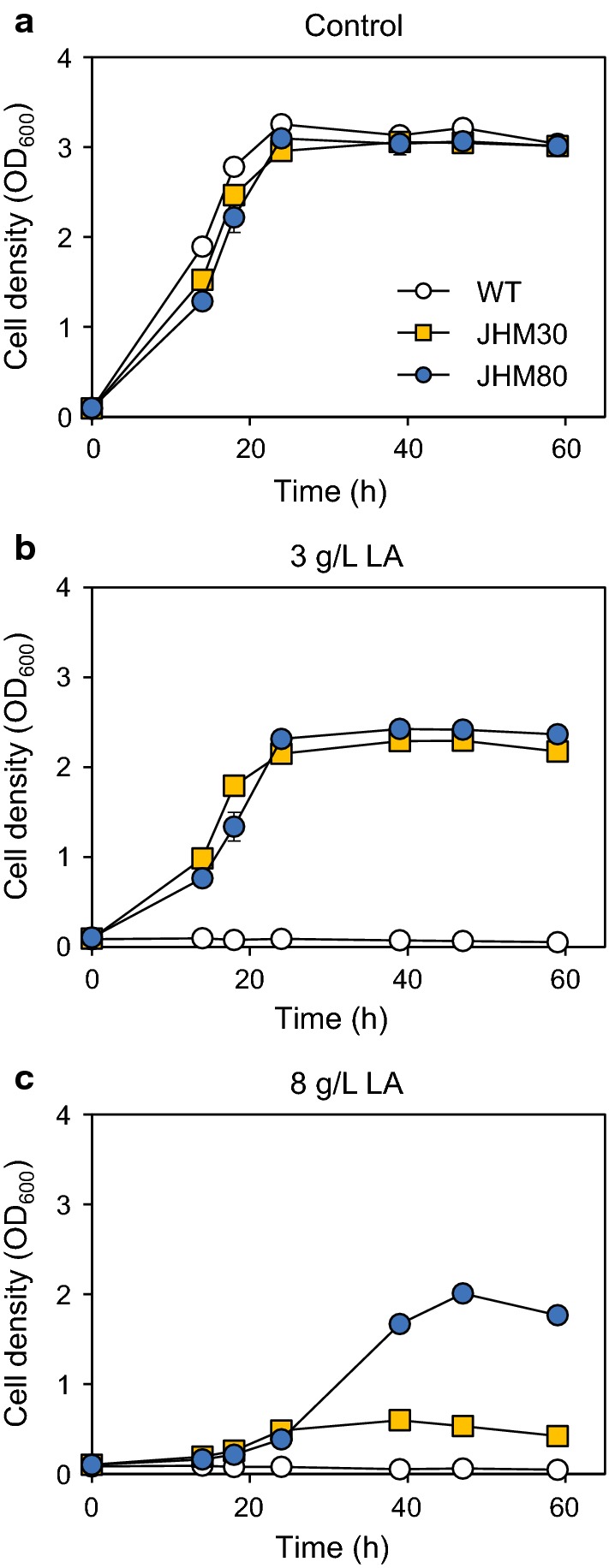
Improved LA tolerance in the evolved strains. Wild-type *Methylomonas* sp. DH-1, JHM30, and JHM80 strains were grown in NMS medium (**a**), or NMS medium containing 3 g/L lactate (**b**) or 8 g/L lactate (**c**). Error bars indicate standard deviations of two independent experiments

Growth inhibition by LA, a weak organic acid, is one of the limiting factors for microbial LA production [25]. Therefore, we first examined the LA tolerance of *Methylomonas* sp. DH-1. The cell growth was severely inhibited by addition of LA, exhibiting low tolerance up to 0.5 g/L LA in the medium neutralized to pH 6.8 (Fig. 1b). To solve this problem of low LA tolerance, we performed adaptive laboratory evolution of *Methylomonas* sp. DH-1 by serially transferring cells to the medium with increasing concentrations of LA from 0.5 g/L to 8.0 g/L. As a result, evolved strains JHM30 and JHM80 were selected, which could survive at 3.0 g/L and 8.0 g/L LA, respectively, during the evolution process. Under normal conditions, these evolved strains and wild type showed comparable growth rates (Fig. 2a). However, in the medium containing 3.0 g/L LA, only the evolved strains could survive (Fig. 2b). In the presence of 8.0 g/L LA, JHM80 showed higher tolerance than JHM30 (Fig. 2c).

### Identification of genes responsible for the enhanced LA tolerance

**Fig. 3:**
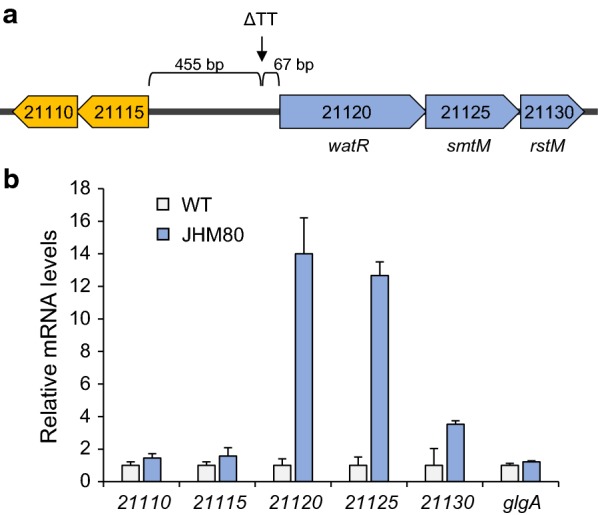
Effects of the intergenic 2-bp (TT) deletion on transcription of the downstream operons. **a** Schematic illustration of the mutation site. **b** The mRNA levels of the indicated genes in wild-type (WT) *Methylomonas* sp. DH-1 and JHM80 were analyzed by qRT-PCR and normalized to the mRNA levels of *mxaF*. The mRNA levels of *glgA* were used as a control

To identify the mutated genes responsible for the enhanced LA tolerance, whole genome sequences of the JHM30 and JHM80 strains were determined. In both JHM30 and JHM80, a deletion of 2 bp (TT) was detected in the intergenic region between the AYM39_21115 and AYM39_21120 genes (Fig. 3a). In JHM80, an additional nonsense mutation was found in the *fliE* gene, where the codon for Gln 49 was changed to a stop codon.

**Fig. 4:**
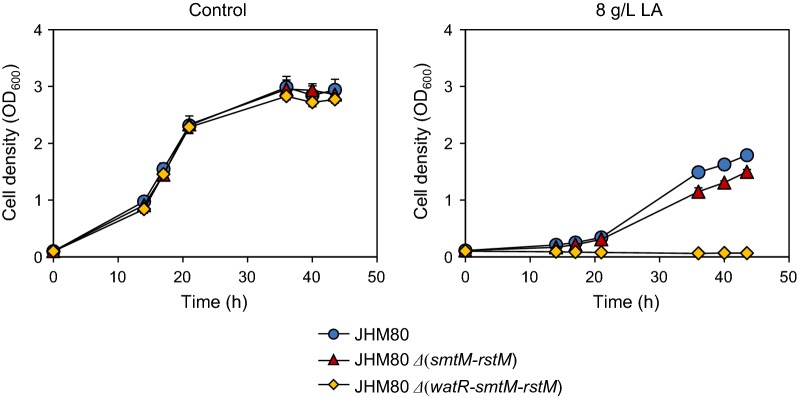
Effect of the *watR* gene deletion on LA tolerance of JHM80. JHM80 and JHM80 with the indicated gene deletions were grown in the absence or the presence of 8.0 g/L LA. Error bars indicate standard deviations of three independent experiments

Since the TT deletion is located in the promoter region, it might affect the transcription of the downstream genes, AYM39_21115 and AYM39_21120, which are parts of operon structures transcribed in opposite directions. Therefore, to investigate the effect of the TT deletion on LA tolerance, we first examined its effect on the transcription of the downstream genes. In comparison with wild type, JHM80 having the TT deletion showed significantly higher expression levels of AYM39_21120 and its downstream genes in the same operon, AYM39_21125 and AYM39_21130 (Fig. 3b). On the other hand, wild type and JHM80 showed similar expression levels of AYM39_21115 and AYM39_21110 genes in the other operon (Fig. 3b). The AYM39_21120 gene (named as *watR*; weak acid tolerance regulator) encodes a LysR-type transcriptional regulator, while the proteins encoded by the AYM39_21125 (named as *smtM*) and AYM39_21130 (named as *rstM*) genes show homology to SAM (S-adenosyl-l-methionine)-dependent methyl transferase and rhodanese related sulfur transferase, respectively (Fig. 3a). To elucidate the role of these up-regulated genes in the LA tolerance of JHM80, we deleted all three genes (*watR*, *smtM*, and *rstM*) or the last two genes (*smtM* and *rstM*) in JHM80 and evaluated the LA tolerance. In the presence of 8 g/L LA, the mutant strain lacking the *smtM* and *rstM* genes showed only slightly lower growth rate than that of JHM80. However, deletion of all three genes abolished the LA tolerance of JHM80, suggesting that the elevated expression of *watR* is mainly responsible for the LA tolerance of JHM80 (Fig. 4).

**Fig. 5:**
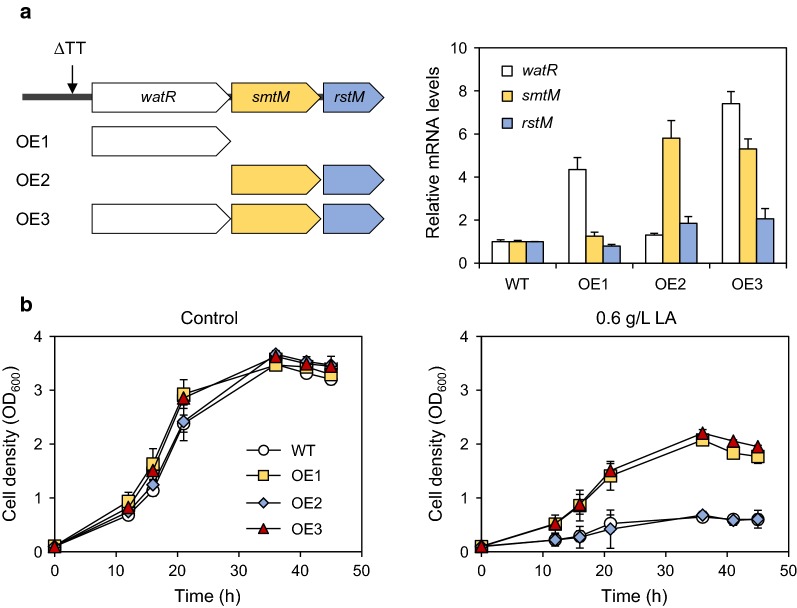
Effect of the *watR* gene overexpression on LA tolerance. **a** The indicated overexpression cassette OE1, OE2, or OE3 was integrated into the genome of wild-type *Methylomonas* sp. DH-1 (WT) and mRNA levels of the overexpressed genes were detected by qRT-PCR. **b** LA tolerance was examined by growing the cells in the absence (Control) or presence of 0.6 g/L LA. Error bars indicate standard deviations of three independent experiments

To further verify the role of the *watR* gene in LA tolerance, we overexpressed the *watR, smtM*, and *rstM* genes in different combinations in the wild-type strain. Using the 500-bp upstream region of the *watR* gene from JHM80 as a promoter, we generated overexpression cassettes for *watR* (OE1), *smtM* and *rstM* (OE2), and all three genes (OE3) (Fig. 5a), and then integrated them into a selected noncoding region of the chromosome. Each integration strain successfully overexpressed the introduced target genes (Fig. 5a). Compared with wild type, cells integrated with the OE1 and OE3 expression cassettes showed higher LA tolerance (Fig. 5b). However, overexpression of OE2 failed to recover the LA sensitivity of the wild-type strain (Fig. 5b). These results further confirm that overexpression of the *watR* gene, but not the *smtM* and *rstM* genes, plays a key role in LA tolerance.

We also examined the effect of *fliE* nonsense mutation identified in JHM80. The *fliE* gene is related to the formation of basal body of flagella [26]. Because the *fliE* mutation was only identified in JHM80, but not in JHM30, we hypothesized that the additional *fliE* mutation might be responsible for the higher LA tolerance of JHM80. Therefore, we deleted the *fliE* gene in JHM30 to mimic the nonsense mutation, but *fliE* deletion did not improve the LA tolerance of JHM30 (Additional file 1: Figure S1). Therefore, the nonsense mutation of *fliE* gene might be not related to LA tolerance. It needs further studies to identify the causal mutations responsible for the higher LA tolerance of JHM80 than JHM30.

### d-Lactate production in JHM80

**Fig. 6:**
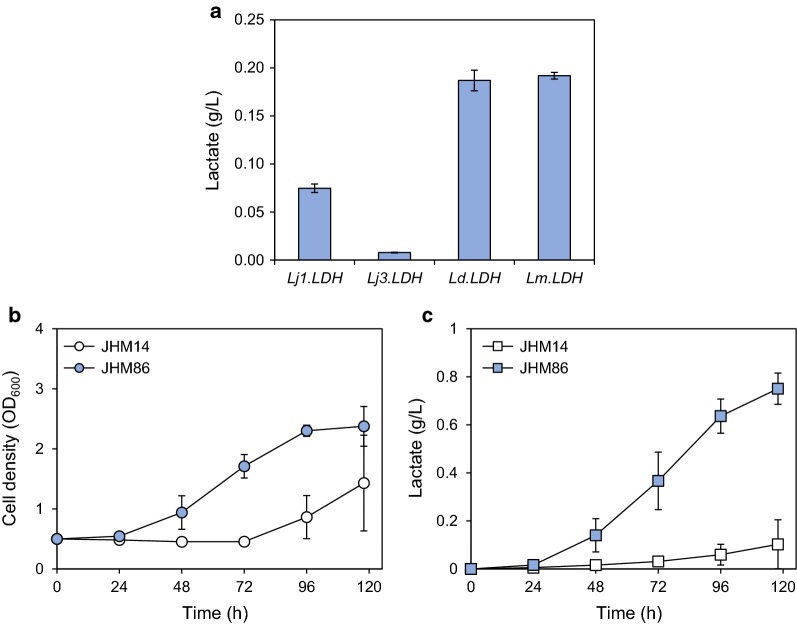
d-LA production by introducing lactate dehydrogenase (LDH) gene in JHM80. **a** The indicated heterologous d form-specific LDH genes from *L. jensenii* (*Lj1.LDH* and *Lj3.LDH*), *L. delbrueckii* subsp. *bulgraricus* ATCC 11842 (*Ld.LDH*), *L. mesenteroides* subsp. *mesenteroides* ATCC 8293 (*Lm.LDH*) were integrated into the genome of JHM80, and LA production levels were detected after growing the cells in NMS medium containing 20% (v/v) methane and 10 μg/mL of kanamycin for 48 h; Wild-type strain integrated with *Lm.LDH* (JHM14) and JHM80 integrated with *Lm.LDH* (JHM86) were grown in a 30-mL serum bottle containing 3-mL NMS medium while feeding 20% (v/v) methane every 24 h. Cell growth (**b**) and LA production (**c**) were monitored during growth. Error bars indicate standard deviations of two independent experiments

*Methylomonas* sp. DH-1 naturally produces pyruvate from methane through the RuMP and EMP pathways. However, it lacks the lactate dehydrogenase (LDH) enzyme, which is necessary for the conversion of pyruvate to lactate (Fig. 1a). To produce d-LA, 4 heterologous D form-specific LDH genes from *L. jensenii* (*Lj1.LDH* and *Lj3.LDH*), *L. delbrueckii* subsp. *bulgaricus* ATCC 11842 (*Ld.LDH*), *L. mesenteroides* subsp. *mesenteroides* ATCC 8293 (*Lm.LDH*) were introduced into the chromosome of JHM80 while deleting the *glgA* gene encoding glycogen synthase. Glycogen is known as a major carbon storage compound in methanotrophs [27, 28]. Upon this genomic integration, the LDH genes were expressed under the control of a native promoter of the *glgBA* operon. JHM80 strains expressing *Lm.LDH* and *Ld.LDH* were more effective in LA production than the strains expressing the other LDH genes, producing 192 mg/L and 187 mg/L LA, respectively, when the cells were grown in NMS medium containing 20% (v/v) methane (Fig. 6a). Therefore, *Lm.LDH* was selected for further experiments.

To further increase d-LA production in the JHM80 strain integrated with the *Lm.LDH* gene (JMH86), methane was fed every 24 h by exchanging the air inside the 30-mL vial with 20% (v/v) methane. Along with periodic methane supply, JHM86 strain produced 750 mg/L d-LA after 118 h (Fig. 6c). On the other hand, wild-type strain integrated with *Lm.LDH* (JHM14) showed very low growth rate and produced only 100 mg/L d-LA (Fig. 6b and c). These results clearly demonstrate that the increase in LA tolerance plays a key role in improving LA production in *Methylomonas* sp. DH-1.

**Fig. 7:**
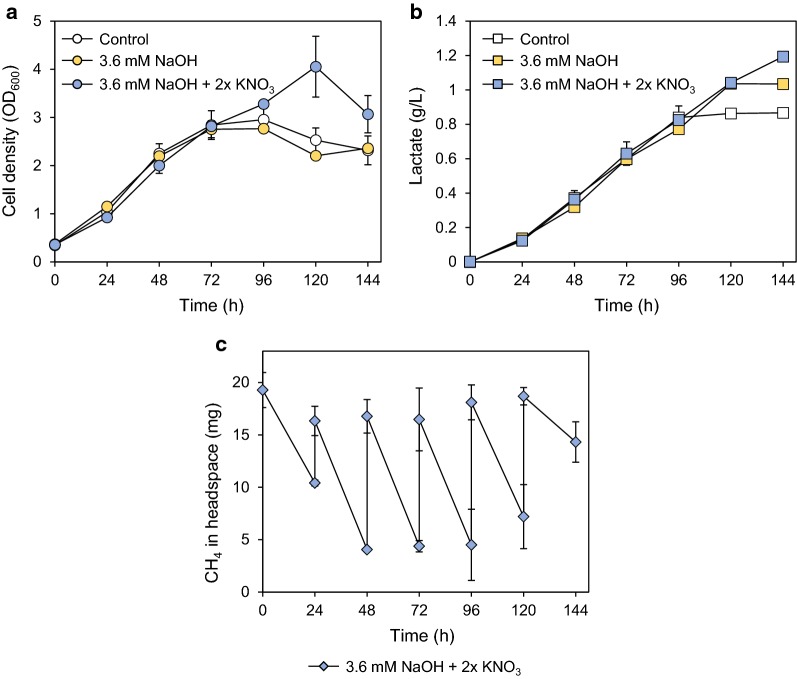
Improvement of LA production by neutralization and medium optimization. JHM86 was grown in a 125-mL flask containing 12.5-mL NMS medium (Control), NMS medium neutralized with 3.6-mM NaOH with or without additional nitrogen source (2× KNO_3_). 20% (v/v) methane was fed every 24 h. Cell growth (**a**) and LA production (**b**) were monitored during growth; **c** Methane consumption in the NMS medium with 3.6 mM NaOH and 2× KNO_3_ was determined by GC chromatography. Methane levels were measured before and after feeding 20% (v/v) methane every 24 h. Any remaining headspace gas was purged before feeding methane. Error bars indicate standard deviations of two independent experiments

In a flask-scale culture supplied with 20% (v/v) methane every 24 h, 860 mg/L d-LA was produced at 144 h (Fig. 7b). Since the medium pH decreased as the accumulation of d-LA during the cultivation, the medium was neutralized by adding 1.2 mM of NaOH at 48 h, 72 h, and 96 h. In this pH-controlled medium, d-LA production increased by about 15% up to 1.04 g/L (Fig. 7b). In spite of the continuous methane supply and neutralization of the medium, JHM86 strain stopped growth after 96 h, which could be due to the depletion of other nutrients such as nitrogen source. In a modified NMS medium containing 2× KNO_3_ (20 mM), JHM86 continued growth up to 120 h, producing 1.19 g/L LA at 144 h with a productivity of 0.008 g/L h (Fig. 7a and b). Under this optimized condition, JHM86 consumed 60.8 mg of methane at 144 h, which was 51% of the supplied methane (Fig. 7c), achieving a d-LA yield of 0.245 g/g CH_4_.

## Discussion

Methanotrophs are promising hosts to produce value-added chemicals from methane [10, 13, 14]. In this context, some efforts have been made to produce LA using methanotrophs [20–22]. In *M*. *buryatense* 5GB1S, the maximum LA titer of 0.8 g/L was achieved even with continuous supply of methane, which coincided with the maximum LA tolerance level of this strain [20], suggesting that LA toxicity could be one of the major limiting factors for high-level LA production. If this is the case, any other metabolic pathway engineering strategies aimed to increase carbon flux to LA might not be successful in improving LA production without solving the problem of LA toxicity.

Here, we developed an LA-tolerant methanotroph strain JHM80 by adaptive laboratory evolution of *Methylomonas* sp. DH-1 and identified that up-regulation of the *watR* gene due to the promoter mutation is partly responsible for the LA tolerance of JHM80. LA tolerance of the wild-type strain was improved by overexpressing the *watR* gene alone, demonstrating successful reverse engineering through the genome analysis of the mutant. The WatR protein shows a homology with the LysR-type transcription factors having a wide range of biological functions as transcriptional activators or repressors [29]. The *smtM* and *rstM* genes, constituting an operon structure with the *watR* gene, were also up-regulated in JHM80, but deletion or overexpression of these genes did not affect LA tolerance. Therefore, other WatR target genes, which can be either up-regulated or down-regulated by the overexpression of *watR*, might be involved in the WatR-mediated LA tolerance. We are currently searching for WatR target genes responsible for LA tolerance. In addition, it needs further studies to elucidate how the transcription level is enhanced by the TT deletion in the promoter. Considering the fact that little is known about transcriptional factors and transcriptional regulations in methanotrophs, further characterization of the WatR regulator could provide important information on the LA tolerance mechanisms in *Methylomonas* sp. DH-1 and possibly in other methanotroph species.

Some methanotrophs, including *M. buryatense* 5GB1 and *M. alcaliphium* 20z^R^ previously used for LA production [20, 22] have their own LDH genes. However, *Methylomonas* sp. DH-1 strain does not have its own LDH gene, which is an advantage to produce optically pure LA. In this study, we generated d-LA-producing strain JHM86 by integrating the *Lm.LDH* gene from *L. mesenteroides* ATCC 8293 into the evolved strain JHM80. Compared with the wild-type strain integrated with the same LDH gene, JHM86 showed about 7.5-fold higher d-LA production, demonstrating the importance of LA tolerance in LA production. In fed-batch flask culture (12.5-mL culture in a 125-mL baffled flask) with medium neutralization and optimization (NMS medium supplemented with 3.6 mM NaOH and 2× KNO_3_), JHM86 produced 1.19 g/L LA with a yield of 0.245 g/g CH_4_ and productivity of 0.01 g/L h. Considering different culture conditions, direct comparison with previous studies might be difficult, but our study showed the highest titer and yield ever reported in LA production from methane. The highest titer reported so far is 0.8 g/L l-LA, which was achieved by high density (OD_600_ ~ 25) culture of engineered *M. buryatense* 5GB1S strain in a bioreactor (3-L culture in a 5-L bioreactor) with continuous methane feeding for 96 h, resulting in a yield of 0.05 g/g CH_4_ and productivity of 0.0084 g/L h [20]. NMS2 medium supplemented with 8× KNO_3_, 2× phosphate buffer, and 4× trace element solution was used in the bioreactor experiments to support high cell growth [20]. In our fed-batch culture conditions, JHM86 produced about 0.8 g/L d-LA at 96 h, even with much lower cell density (OD_600_ ~ 3). In another study using small-scale fed-batch culture (2-mL culture in a 27-mL Hungate tube) and neutralized MMS2 medium replacing nitrate with ammonium as a nitrogen source, engineered *M. buryatense* 5GB1 strain (OD_600_ ~ 1) produced about 0.5 g/L l-LA at 72 h [21].

JHM80 could survive in the presence of 8 g/L LA at pH 6.8, but the JHM80-derived LA-production strain JHM86 showed reduced growth rate although its LA production level was below the maximum tolerance level (compare Fig. 2 and Fig. 7), suggesting that intracellular production of LA is still toxic in JHM80 strain. Under our LA tolerance test conditions of pH 6.8, LA (p*K*_*a*_ = 3. 86) mainly exists as a dissociated anion form, which has limited permeability to the plasma membrane unlike the acid form that can freely diffuse across the membrane [30, 31]. Therefore, cellular tolerance level to lactate produced inside might be different from that observed by external treatment of LA. Lactate production might inhibit cell growth through intracellular acidification and various metabolic effects of acid anion as suggested in other microorganisms [25, 32]. In addition, redirection of the pyruvate flux to lactate might reduce the downstream metabolic pathways including the TCA cycle, thus affecting cell growth. Therefore, further elucidation of LA tolerance mechanisms and metabolic pathways might be necessary to improve LA production. In addition, LA production could be further enhanced by metabolic pathway engineering including the regulation of LDH gene expression levels, elimination of competing pathways, and optimization of fermentation medium and conditions.

## Conclusions

Due to the unique ability of utilizing methane as a sole feedstock, methanotrophs are considered as promising hosts for the bioconversion of methane to value-added chemicals. By integrating heterologous LDH gene into the genome of JHM80, an LA-tolerant strain generated by adaptive laboratory evolution of *Methylomonas* sp. DH-1, we developed a strain JHM86 that can effectively produce d-LA. JHM86 produced 1.19 g/L d-LA with a yield of 0.245 g/g CH_4_ and productivity of 0.01 g/L h in fed-batch culture with periodic methane feeding. Furthermore, overexpression of the *watR* gene encoding a LysR-type transcription factor was identified to be responsible for the increased LA tolerance of JHM80.

## Methods

### Strains and culture conditions

All strains used in this study are listed in Table 1. *Methylomonas* sp. DH-1 (KCTC13004BP) was used as a parental strain. *Methylomonas* sp. DH-1 was cultured in nitrate mineral salts (NMS) medium [33] supplemented with 20% (v/v) methane at 30 °C with shaking at 170 rpm. Methanotroph strains were grown in 3-mL NMS medium in a 30-mL serum bottle capped with a butyl rubber stopper or in 12.5-mL NMS medium in a 125-mL baffled flask sealed with rubber type screw cap. For LA production, 10 μg/mL of kanamycin was added to the medium. For repeated methane feeding, headspace of the culture was purged and 20% (v/v) methane was added every 24 h.

**Table 1:** Strains used in this study

Strain	Genotype	References
*Methylomonas* sp. DH-1	Wild-type strain	[23]
JHM11	DH-1 AYM39_05845::P_*watR*_ (*ΔTT*)-*watR*-T_*rrnB*_ (OE1)::AYM39_05850	This study
JHM12	DH-1 AYM39_05845::P_*watR*_ (*ΔTT*)-*smtM, rstM*-T_*rrnB*_ (OE2)::AYM39_05850	This study
JHM13	DH-1 AYM39_05845:: P_*watR*_ (*ΔTT*)-*watR, smtM, rstM*-T_*rrnB*_ (OE3)::AYM39_05850	This study
JHM14	DH-1 *ΔglgA::Lm.LDH*-*Kan*^*R*^	This study
JHM30	Evolved strain from DH-1	This study
JHM31	JHM30 *ΔfliE::Kan*^*R*^	This study
JHM80	Evolved strain from DH-1	This study
JHM81	JHM80 *Δ*(*smtM*-*rstM*)*::Kan*^*R*^	This study
JHM82	JHM80 *Δ(watR*-*smtM*-*rstM)::Kan*^*R*^	This study
JHM83	JHM80 *ΔglgA::Lj1.LDH*-*Kan*^*R*^	This study
JHM84	JHM80 *ΔglgA::Lj3.LDH*-*Kan*^*R*^	This study
JHM85	JHM80 *ΔglgA::Ld.LDH*-*Kan*^*R*^	This study
JHM86	JHM80 *ΔglgA::Lm.LDH*-*Kan*^*R*^	This study

### Plasmid construction

Plasmid and primers used in this study are listed in Table 2 and Additional file 2: Table S1. The 1-kb upstream DNA fragment of the *glgA* (AYM39_03770) gene (U_*glgA*_), LDH gene from *Leuconostoc mesenteroides* subsp. *mesenteroides* ATCC8293 (*Lm.LDH*), and *rrnB* terminator (T_*rrnB*_) from *Escherichia coli* were prepared by PCR amplification from *Methylomonas* sp. DH-1 genomic DNA, p425-ADH-*Lm.ldhA* [3], and *E. coli* DH-5α genomic DNA, respectively. These PCR products were cloned between the *Nhe*I and *Eco*RI sites of pCM184 [34] by using AccuRapid™ Cloning Kit (Bioneer, Korea), generating pU_glgA_-Lm.LDH. The 1-kb downstream DNA fragment of the *glgA* gene (D_*glgA*_) was amplified by PCR and cloned between the *Apa*I and *Sac*I sites of pCM184, generating pD_glgA_. The DNA fragments encoding pBR322 replication origin with or without ampicillin resistance gene (*Amp*^*R*^), U_*glgA*_-*Lm.LDH*-T_*rrnB*_, and kanamycin resistance gene (*Kan*^*R*^) with D_*glgA*_ were prepared by PCR amplification from pCM184, pU_glgA_-Lm.LDH, and pD_glgA_, respectively, and ligated using AccuRapid™ Cloning Kit, generating pDel-glgA-Lm.LDH (with *Amp*^*R*^) and pDel2-glgA-Lm.LDH (without *Amp*^*R*^). For the integration of other heterologous LDH genes, LDH genes from *Lactobacillus jensenii* (*Lj1.LDH* and *Lj3.LDH*) and *Lactobacillus delbrueckii* subsp. *bulgaricus* ATCC 11842 (*Ld.LDH*) were prepared by PCR amplification from p425ADH-*Lj.ldh1*, p425ADH-*Lj.ldh3*, and p425ADH-*Ld.ldhA* [3], and cloned between the *Nde*I and *Mlu*I sites of pDel-glgA-Lm.LDH, resulting in pDel-glgA-Lj1.LDH, pDel-glgA-Lj3.LDH, and pDel-glgA-Ld.LDH, respectively.

**Table 2:** Plasmids used in this study

Plasmid	Description	References
pCM184	*Amp*^*R*^, *Kan*^*R*^, *Tet*^*R*^; broad-host range allelic exchange vector	[34]
pDel-glgA-Lm.LDH	pCM184-U_*glgA*_-[*Lm.LDH*-T_*rrnB*_-*Kan*^*R*^]-D_*glgA*_	This study
pDel-glgA-Lj1.LDH	pCM184-U_*glgA*_-[*Lj1.LDH*-T_*rrnB*_-*Kan*^*R*^]-D_*glgA*_	This study
pDel-glgA-Lj3.LDH	pCM184-U_*glgA*_-[*Lj3.LDH*-T_*rrnB*_-*Kan*^*R*^]-D_*glgA*_	This study
pDel-glgA-Ld.LDH	pCM184-U_*glgA*_-[*Ld.LDH*-T_*rrnB*_-*Kan*^*R*^]-D_*glgA*_	This study
pDel2-glgA-Lm.LDH	pDel-glgA-Lm.LDH without *Amp*^*R*^	This study
pIns	Plasmid containing [U_Ins_-T_*rrnB*_-*Kan*^*R*^-D_Ins_] cassette for integration into noncoding region between AYM39_05845 and AYM39_05850	This study
pIns-mW	pIns-[P_*watR*_ (*ΔTT*)-*watR*-T_*rrnB*_]	This study
pIns-mSR	pIns-[P_*watR*_ (*ΔTT*)- *smtM, rstM*-T_*rrnB*_]	This study
pIns-mWSR	pIns-[P_*watR*_ (*ΔTT*)-*watR, smtM, rstM*-T_*rrnB*_]	This study
pDel2-fliE	pDel2-U_*fliE*_-[T_*rrnB*_-*Kan*^*R*^]-D_*fliE*_	This study
pDel2-SR	pDel2-U_*smtM*_-[T_*rrnB*_-*Kan*^*R*^]-D_*rstM*_	This study
pDel2-WSR	pDel2-U_*watR*_-[T_*rrnB*_-*Kan*^*R*^]-D_*rstM*_	This study

To construct plasmid for DNA integration into a noncoding region of *Methylomonas* sp. DH-1 chromosome, two consecutive DNA fragments between AYM39_05845 and AYM39_05850 (U_Ins_ and D_Ins_) were amplified by PCR and sequentially cloned into the *Not*I/*Spe*I and *Apa*I/*Sac*I sites of pDel2-glgA-Lm.LDH, generating pIns. The *watR*, *smtM*-*rstM*, and *watR*-*smtM*-*rstM* operon genes were prepared with the 500-bp upstream sequence including a deletion of 2 bp (TT) [P_*watR*_ (*ΔTT*)] by PCR amplification or overlap extension PCR using JHM80 genomic DNA as a template, and then cloned between the *Spe*I and *Kpn*I sites of pIns plasmid, resulting in pIns-mW, pIns-mSR, and pIns-mWSR, respectively.

To construct plasmid for gene deletion, U_Ins_ and D_Ins_ sequences in pIns plasmid were replaced with 1-kb upstream and downstream sequences of the target genes using *Not*I/*Spe*I and *Apa*I/*Sac*I sites, respectively.

### Genetic manipulation of *Methylomonas* sp. DH-1

Gene deletion or integration of DNA in *Methylomonas* sp. DH-1 was achieved by homologous recombination between the chromosome and plasmid vector containing 1 kb each of upstream and downstream regions of the target integration site. Proper integration of the target DNA or gene deletion was confirmed by PCR analysis using confirmation primers. Plasmid DNA was introduced by electroporation as previously reported with some modifications [14, 15]. OD_600_ of 0.2 cells were spread onto a NMS plate and cultured for 3 days while supplying 30% methane. The biomass was harvested from the plate with distilled water and then transferred to 1.5-mL microcentrifuge tubes. After centrifugation at 14,000 rpm for 2 min, cells were washed twice with distilled water. 50 μL of cell suspension was mixed gently with 3-μL DNA and the mixture was transferred to an ice-cold 2-mm-gap cuvette (Bio-Rad, USA). Electroporation was performed using a Gene Pulser II system (Bio-Rad, USA) at preprogrammed Ec2 setting. Immediately after electric shock, cells were resuspended with 1 mL of NMS medium and then transferred to 30-mL bottle supplied with additional 2 mL of medium and 20% (v/v) methane. After overnight incubation in a shaking incubator, cells were harvested by centrifugation at 14,000 rpm for 2 min, and then spread onto a selective NMS plate containing 10 μg/mL of kanamycin.

### Laboratory adaptive evolution

To develop LA-tolerant mutants of *Methylomonas* sp. DH-1, cells were adapted to LA by growing in NMS medium with gradually increasing concentrations of LA from 0.5 g/L to 8 g/L during 35 subcultures. The pH of the NMS medium containing LA was adjusted to 6.8 with NaOH.

### Whole genome sequencing analysis

Genomic DNA of *Methylomonas* sp. DH-1 and evolved strains JHM30 and JHM80 was isolated using a bacteria genomic DNA extraction kit (iNtRON Biotechnology, Korea). DNA libraries were generated using a Truseq Nano DNA LT kit (Illumina, USA) and sequenced using PE 2× 300-Miseq (Illumina, USA). Mutated DNA sequences in JHM30 and JHM80 were analyzed as described previously [35].

### Quantitative reverse transcription PCR (qRT-PCR)

*Methylomonas* sp. DH-1 and JHM80 cells were cultured in 12.5-mL NMS medium supplied with 20% (v/v) methane in a 125-mL flask for 16 h. Total RNA was extracted using RNeasy Mini kit (Qiagen, USA) according to the manufacturer’s instructions. The relative amount of mRNA was determined by quantitative reverse transcription PCR (qRT-PCR) as previously described [36] with minor modifications. 1 μg of total RNA was used for reverse transcription in a 25-μL reaction volume containing 200 unit of M-MLV reverse transcriptase (Thermo Fishers Scientific, USA), 0.1 μg of random hexamer, and 2 μL each of 10-mM dNTPs. After incubation at 25 °C for 10 min and 42 °C for 60 min, reverse transcription was terminated by heating at 70 °C for 10 min. For qRT-PCR analysis, 1 μL of cDNA (diluted 1:20) was amplified by SYBR Green I maser mix (Roche-Applied Science, USA) using 0.75 pmol each of gene-specific primers with 45 cycles of 95 °C for 20 s, 55 °C for 20 s, and 72 °C for 20 s on a Lightcycler 480 II System (Roche Applied Science, USA). Primer sequences used for qRT-PCR are listed in Additional file 2: Table S1.

### Analytical methods

Cell growth was monitored by measuring optical densities at 600 nm using Multiskan GO spectrophotometer (Thermo Fishers Scientific, USA). To determine the concentrations of metabolites, 300 μL of culture supernatants were filtered through 0.22-μm syringe filter and analyzed by high-performance liquid chromatography (HPLC) as described previously [37]. To measure the amount of methane consumed during the repeated methane feeding, methane levels were measured before and after the methane feeding. 300 μL of gas mixture collected from headspace of flask was analyzed using gas chromatography (Younglin 6500GC, YL instruments, Korea) equipped with a molecular sieve 13× packed column (13047-U, SUPELCO, USA) and Porapak N packed column (13052-U, SUPELCO, USA) with argon at a flow rate of 15 mL/min as a carrier gas. The analytes were detected by thermal conductivity detector maintained at 120 °C.

## Supplementary information

**Additional file 1: Figure S1.** Effect of the *filE* gene deletion on LA tolerance of JHM30. JHY30, JHY31 (JHM30 *ΔfliE::Kan*^*R*^), JHM80 were grown in the absence or the presence of 8.0 g/L LA. Error bars indicate standard deviations of three independent experiments.

**Additional file 2: Table S1.** Primers used in this study.

## Data Availability

All data generated or analyzed during this study are included in this published article and its additional files.

## Abbreviations

CBB: Calvin–Benson–Bassham
EMP: Embden–Meyerhof–Parnas
EDD: Entner–Doudoroff
HPLC: high-performance liquid chromatography
LA: lactic acid
LDH: lactate dehydrogenase
MDH: methanol dehydrogenase
PLA: polylactic acid
MMO: methane monooxygenase
qRT-PCR: quantitative reverse transcription PCR
RuMP: ribulose monophosphate
SAM: *S*-adenosyl-l-methionine
