# Supplementary material for: Efficient production of d-lactate from methane in a lactate-tolerant strain of Methylomonas sp. DH-1 generated by adaptive laboratory evolution
Source: Biotechnol Biofuels. 2019 Sep 30;12:234. doi: 10.1186/s13068-019-1574-9 (PMC6767647; doi:10.1186/s13068-019-1574-9)
Supplement: Supplementary file 1 — Additional file 1: Figure S1. Effect of the filE gene deletion on LA tolerance of JHM30. JHY30, JHY31 (JHM30 ΔfliE::KanR), JHM80 were grown in the absence or the presence of 8.0 g/L LA. Error bars indicate standard deviations of three independent experiments. [file 13068_2019_1574_MOESM1_ESM.docx]

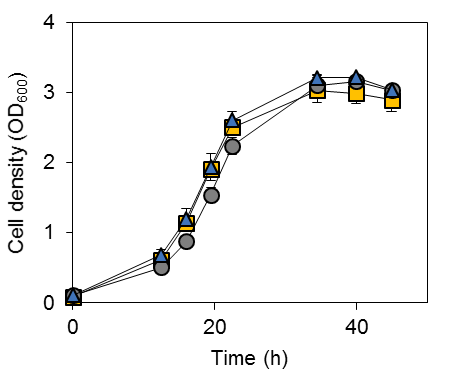

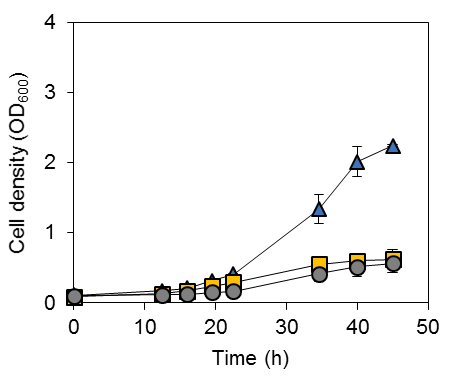


Control

8 g/L LA

**a**

**b**

JHM30

JHM30 *Δ fliE*

JHM80

**Fig. S1.** Effect of the *filE* gene deletion on LA tolerance of JHM30. HY30, JHY31 (JHM30 *ΔfliE::Kan^R^*), JHM80 were grown in the absence or the presence of 8.0 g/L LA. Error bars indicate standard deviations of three independent experiments.
