# Supplementary material for: Efficient production of d-lactate from methane in a lactate-tolerant strain of Methylomonas sp. DH-1 generated by adaptive laboratory evolution
Source: Biotechnol Biofuels. 2019 Sep 30;12:234. doi: 10.1186/s13068-019-1574-9 (PMC6767647; doi:10.1186/s13068-019-1574-9)
Supplement: Supplementary file 2 — Additional file 2: Table S1. Primers used in this study. [file 13068_2019_1574_MOESM2_ESM.docx]

**Table S1. Primers used in this study**

| **Primers** | **Sequence (5’-3’)** | |
| --- | --- | --- |
| Primers used for confirmation of gene manipulation | | |
| c_glgA F | | CAACTACAGCCGTAACGAAG |
| c_watR F | | CAGCACATCGGCTTCTTTGT |
| c_smtM F | | CGTTAAGTTGCCGCACACTA |
| c_fliE F | | ACTGTATGCTTTGGCCGGCA |
| c_Univ-Ins F | | GCAATCAAAATCTGCTCCGC |
| c_Univ R | | ACGTGGTTAATTAATTTGTCCTACTCAGGAGA |
| Primers used for qRT-PCR | | |
| q_15615 (mxaF) F | | CCGCTTTCAACATCAAGGAC |
| q_15615 (mxaF) R | | GCTGTCGTAAGCGTACCAGC |
| q_21110 F | | TCCGCATTTATTGGTGGTGC |
| q_21110 R | | TGCTGGAAACTTCGCCTTCC |
| q_21115 F | | AGCAGCGCAAACAACAGTCG |
| q_21115 R | | CTAGTTCCTGGTGCGCCAAC |
| q_21120 (watR) F | | TATCTGGAACGCTGCCAGCA |
| q_21120 (watR) R | | TGACCGCCTTTCAGCACCAT |
| q_21125 (smtM) F | | GGCTAAGCCTGAGCGTCAAC |
| q_21125 (smtM) R | | GGGCCGTGTTGGTCAAGCTT |
| q_21130 (rstM) F | | AATCCCAACGCCGTGCTGAT |
| q_21130 (rstM) R | | ACAGAACGTTGTCCGCTGCG |
| q_03770 (glgA) F | | TGGAAGGCAAACAGGCCAAT |
| q_03770 (glgA) R | | GTACTCTATGCTCTTGTCGC |
